# Combinations of β-Lactam or Aminoglycoside Antibiotics with Plectasin Are Synergistic against Methicillin-Sensitive and Methicillin-Resistant *Staphylococcus aureus*

**DOI:** 10.1371/journal.pone.0117664

**Published:** 2015-02-18

**Authors:** Yanmin Hu, Alexander Liu, James Vaudrey, Brigita Vaiciunaite, Christiana Moigboi, Sharla M. McTavish, Angela Kearns, Anthony Coates

**Affiliations:** 1 Institute for Infection and Immunity, St George’s, University of London, London, United Kingdom; 2 University of Oxford Centre for Clinical Magnetic Resonance Research, University of Oxford, Oxford, United Kingdom; 3 Antimicrobial Resistance and Healthcare Associated Infections Reference Unit, Public Health England, Colindale, London, United Kingdom; Kliniken der Stadt Köln gGmbH, GERMANY

## Abstract

Bacterial infections remain the leading killer worldwide which is worsened by the continuous emergence of antibiotic resistance. In particular, methicillin-sensitive (MSSA) and methicillin-resistant *Staphylococcus aureus* (MRSA) are prevalent and the latter can be difficult to treat. The traditional strategy of novel therapeutic drug development inevitably leads to emergence of resistant strains, rendering the new drugs ineffective. Therefore, rejuvenating the therapeutic potentials of existing antibiotics offers an attractive novel strategy. Plectasin, a defensin antimicrobial peptide, potentiates the activities of other antibiotics such as β-lactams, aminoglycosides and glycopeptides against MSSA and MRSA. We performed *in vitro* and *in vivo* investigations to test against genetically diverse clinical isolates of MSSA (n = 101) and MRSA (n = 115). Minimum inhibitory concentrations (MIC) were determined by the broth microdilution method. The effects of combining plectasin with β-lactams, aminoglycosides and glycopeptides were examined using the chequerboard method and time kill curves. A murine neutropenic thigh model and a murine peritoneal infection model were used to test the effect of combination *in vivo*. Determined by factional inhibitory concentration index (FICI), plectasin in combination with aminoglycosides (gentamicin, neomycin or amikacin) displayed synergistic effects in 76-78% of MSSA and MRSA. A similar synergistic response was observed when plectasin was combined with β-lactams (penicillin, amoxicillin or flucloxacillin) in 87–89% of MSSA and MRSA. Interestingly, no such interaction was observed when plectasin was paired with vancomycin. Time kill analysis also demonstrated significant synergistic activities when plectasin was combined with amoxicillin, gentamicin or neomycin. In the murine models, plectasin at doses as low as 8 mg/kg augmented the activities of amoxicillin and gentamicin in successful treatment of MSSA and MRSA infections. We demonstrated that plectasin strongly rejuvenates the therapeutic potencies of existing antibiotics *in vitro* and *in vivo*. This is a novel strategy that can have major clinical implications in our fight against bacterial infections.

## Introduction

Bacterial infection remains a leading killer worldwide [1] and antibiotic resistance continues to plague the effective control of this pandemic health problem. Currently, resistance to marketed antibiotics is prevalent and most of the existing classes are now redundant for an increasing number of bacterial species [2,3] and the number of new antibiotics reaching the market annually is unable to keep up with the development of resistance [4]. *Staphylococcus aureus* is one of the commonest pathogens in both hospital and community acquired infections. Methicillin-sensitive (MSSA) and methicillin-resistant *S. aureus* (MRSA) cause significant morbidity and mortality and are associated with an array of life-threatening infections including surgical site infections, bacteremia, pneumonia and catheter-associated infections [5,6]. There is a very limited antimicrobial armamentarium to treat infections caused by MRSA, of which vancomycin and linezolid are the major agents but resistance to these agents is emerging [7,8]. It may be argued that the traditional strategy of antibiotic discovery is flawed because rapid bacterial selection inevitably leads to resistance development within only a few years post market release. Moreover, the drug discovery process itself is arduous and costly, meaning the emergence of a large group of effective antibiotics within a short time period is almost impossible. Therefore, a different approach is needed to replenish our armamentarium against resistant bacteria and the most promising strategy is to restore the therapeutic potencies of existing antibiotics [9–11].

Weakening the cell envelope and increasing cellular permeability are two effective therapeutic strategies to enhance the effectiveness of some antibiotics [12–14]. This is exploited by compounds that target the bacterial cell membrane or cell wall [12–14]. In particular, peptides have been shown to be potential enhancers to augment the activities of other antibiotics [15–17]. Plectasin, a defensin derived from *Pseudoplectania nigrella*, strongly synergises with other antibiotics [16]. Plectasin alone possesses antimicrobial activity and is bactericidal against many Gram-positive bacteria including drug resistant Streptococci and Staphylococci [18]. It acts by binding to the pyrophosphate moiety of lipid II in the bacterial cell wall, thus leading to the rapid killing of the target bacteria [19]. A newer version of plectasin, NZ2114, has shown a strong therapeutic efficacy against *S. aureus* and *Streptococcus pneumoniae* in various different animal infection models [20–22]. Furthermore, plectasin is stable in serum, exhibits a long half-life *in vivo* and is non-toxic in both cell culture and animals [18,23]. The pharmacokinetic and pharmacodynamic property of plectasin *in vitro* and *in vivo* makes it an ideal adjuvant compound to enhance the activity of other clinically used antibiotics.

In this study, we aimed to test the *in vitro* and *in vivo* activity of plectasin in combination with a number of antibiotics, namely penicillins (penicillin, amoxicillin, and flucloxacillin), aminoglycosides (gentamicin, neomycin and amikacin) and a glycopeptide (vancomycin) against clinical isolates of MSSA and MRSA.

## Results

### *In vitro* susceptibility of plectasin against MSSA and MRSA

The MIC of plectasin NZ2114 was determined against 101 MSSA and 115 MRSA. As shown in Table 1, the MIC range for plectasin was 0.5 to 4 mg/L for MSSA and 0.5 to 8 mg/L for MRSA. The MIC_50_ and MIC_90_ of the isolates were 2 and 4 mg/L, respectively.

**Table 1:** Minimal inhibitory concentration of plectasin against clinical isolates of MSSA and MRSA.

		MIC^a^ (mg/L)		
Bacterial strains	Number of isolates	MIC range	MIC_50_	MIC_90_
MSSA	101	0.5–4	2	4
MRSA	115	0.5–8	2	4

a MIC_50_ and MIC_90_ 50% and 90% indicate that MIC values at which 50 and 90% of the isolates were inhibited, respectively.

### Chequerboard analysis of combination effects

The activity of plectasin in combination with (i) beta-lactam antibiotics (penicillin, amoxicillin and flucloxacillin), (ii) aminoglycosides (gentamicin, neomycin and amikacin) or (iii) vancomycin was determined using the broth microdilution chequerboard assay against the same test panel of MSSA and MRSA strains. The values of the FIC index for these combinations are shown in Table 2. The combination of plectasin with the β-lactams tested demonstrated synergistic activities for 87–89% of test isolates. Notably, significant reductions in β-lactam MICs were observed, including the majority of the MRSAs tested (S1 and S2 Tables). Similarly, the combination of plectasin with the aminoglycosides tested showed synergistic activities against 76–78% of test isolates. The MIC of the aminoglycosides in the test strains was significantly reduced with the presence of plectasin (S1 and S2 Tables). In addition, a 4- to 32-fold reduction in the plectasin MIC was observed with both antibiotic classes (data not shown). In contrast, there was no interaction between plectasin and vancomycin. No antagonism was observed among any of the combinations tested.

**Table 2:** Combination actives of plectasin with ß-lactams, aminoglycosides and vancomycin.

			Total no. (%) of strains detected when plectasin combined with						
Strains	Combination activity	FICI	amoxicillin	penicillin	flucloxacillin	gentamicin	neomycin	amikacin	vancomycin
MSSA	synergy	≤ 0.5	90 (89.1%)	89 (88.1%)	88 (87.1%)	79 (78.2%)	76 (75.3%)	77 (76.2%)	0
	no interaction	0.56–1	11 (10.9%)	12 (11.9%)	13 (12.9%)	22 (21.8%)	25 (24.7%)	24 (23.8%)	101 (100%)
	antagonism	>4	0	0	0	0	0	0	0
MRSA	synergy	≤ 0.5	101 (87.8%)	102 (88.7%)	102 (88.7%)	89 (77.4%)	88 (76.5%)	88 (76.5%)	0
	no interaction	0.56–1	14 (12.2%)	13 (11.3%)	13 (11.3%)	26 (22.6%)	27 (23.5%)	27 (23.5%)	115 (100%)
	antagonism	>4	0	0	0	0	0	0	0

### *Spa* typing of MSSA and MRSA

*Spa* typing data showed the MSSAs were more heterogeneous genotypically than the MRSAs studied, belonging to 16 and 6 different MLST-CCs respectively (Table 3). The majority of MRSAs (109; 94.8%) were representatives of the EMRSA-15 and-16 lineages (CC22 and CC30) which have remained the dominant HA-MRSA clones seen in the UK for over two decades.

**Table 3:** *Spa* types of MSSA and MRSA isolates.

Strains	MLST-CC (inferred)	Spa types (n)
MSSA	1	t127 (4), t189 (1), t273 (1), t1778 (1), t14364 (1)
	5	t179 (1), t242 (1), t442 (1), t502 (1), t1305 (1), t14367 (1)
	7	t091 (1)
	8	t008 (5), t024 (3), t118 (1), t334 (1), t2067 (1), t14363 (1)
	12	t160 (1), t213 (1), t771 (1), t888 (1), t2133 (1)
	15	t084 (3), t328 (1), t346 (1), t393 (1), t491 (1)
	20	t164 (1)
	22	t005 (3), t032 (3), t709 (1), t891 (1), t1370 (1), t6642 (3), t10276 (1)
	25	t1149 (2), t2313 (1)
	30	t012 (2), t018 (3), t019 (1), t021 (5), t089 (1), t136 (1), t318 (4), t817 (1), t14366 (2)
	45	t015 (4), t026 (1), t040 (1), t073 (1), t563 (1), t848 (1), t2195 (1), t4982 (1), t6243 (1), t14365 (1)
	59	t216 (1)
	80	t044 (1)
	97	t267 (7)
	101	t056 (1)
	121	t171 (1), t645 (1)
MRSA	1	t2478 (1)
	5	t002 (1), t311 (1)
	8	t008 (1), t037 (1)
	22	t020 (3), t022 (4), t025 (1), t032 (55), t379 (2), t578 (1), t611 (1), t756 (1), t1249 (2), t1370 (1), t1467 (1), t2006 (1), t3178 (2), t12788 (1)
	30	t018 (29), t021 (1), t253 (3)
	101	t280 (1)

### Time kill analysis of the combinations against MSSA and MRSA

**Fig 1:**
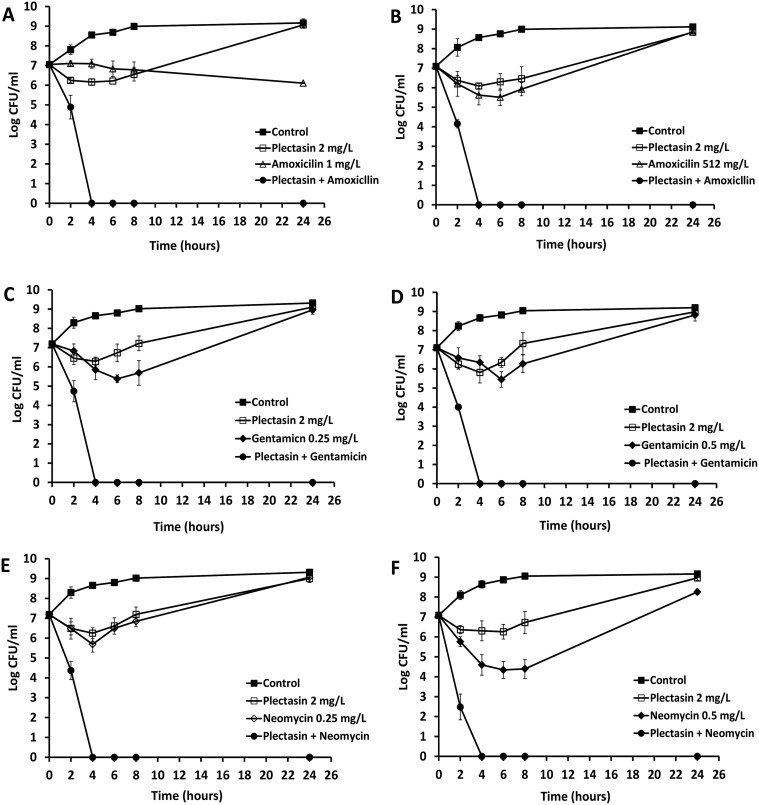
Time-kill analysis showing the effects of plectation in combination with amoxicillin, gentamicin and neomycin against log phase MSSA and MRSA. The peptide and antibiotics alone or each combined with plectasin were added to the log-phase cultures and CFU counts were carried out at different time points. Combination of plectasin (2 mg/L) and amoxicillin (1 mg/L or 512 mg/L) against MSSA (A) and MRSA (B). Combination of plectasin (2 mg/L) and gentamicin (0.25 and 0.5 mg/L) against MRSA (C) and MRSA (D). Combination of plectasin (2 mg/L) and neomycin (0.25 and 0.5 mg/L) against MSSA (E) and MRSA (F). Results shown are the mean of three independent experiments.

The synergistic activities of plectasin with amoxicillin, gentamicin and neomycin were examined with a time kill assay against log-phase MSSA and MRSA with 5 of each MSSA and MRSA which showed FIC index <0.5 for each drug combination. A range of different concentrations for the antibiotics and plectasin was determined according to the chequerboard results. These concentrations were used for each treatment, either with a single agent or in combination. The combinations with most effective synergistic activity against log-phase bacteria are shown. In Fig. 1, plectasin at the MIC value of 2 mg/L killed the bacteria initially, but regrowth was observed after 4 hours of incubation. Amoxicillin at a concentration of 1 mg/L (MIC) inhibited the MSSA growth (A) but had poor efficacy against MRSA at the MIC concentration 512 mg/L (B). However, in combination with plectasin, the initial inoculum was reduced by 100% at about 4 hours post treatment for both MSSA and MRSA (A and B). Gentamicin (C and D) and neomycin (E and F) at a concentration of 0.25 or 0.5 mg/L (MIC) showed bactericidal activities initially against MSSA and MRSA, respectively, followed by a regrowth reaching a peak at 24 hours. The regrowth may be attributable to drug degradation or microorganism adaptation [24,25]. However, in combination with plectasin, there was a 100% reduction in viable counts at 4 hours against both MSSA and MRSA for all combinations tested. The CFU counts remained at zero for 24 hours and no regrowth was observed at 48 hours (data not shown). The time kill assay demonstrated that there was a significant synergistic activity between plectasin and amoxicillin, gentamicin or neomycin.

### *In vivo* combination activities of plectasin with amoxicillin and gentamicin

**Fig 2:**
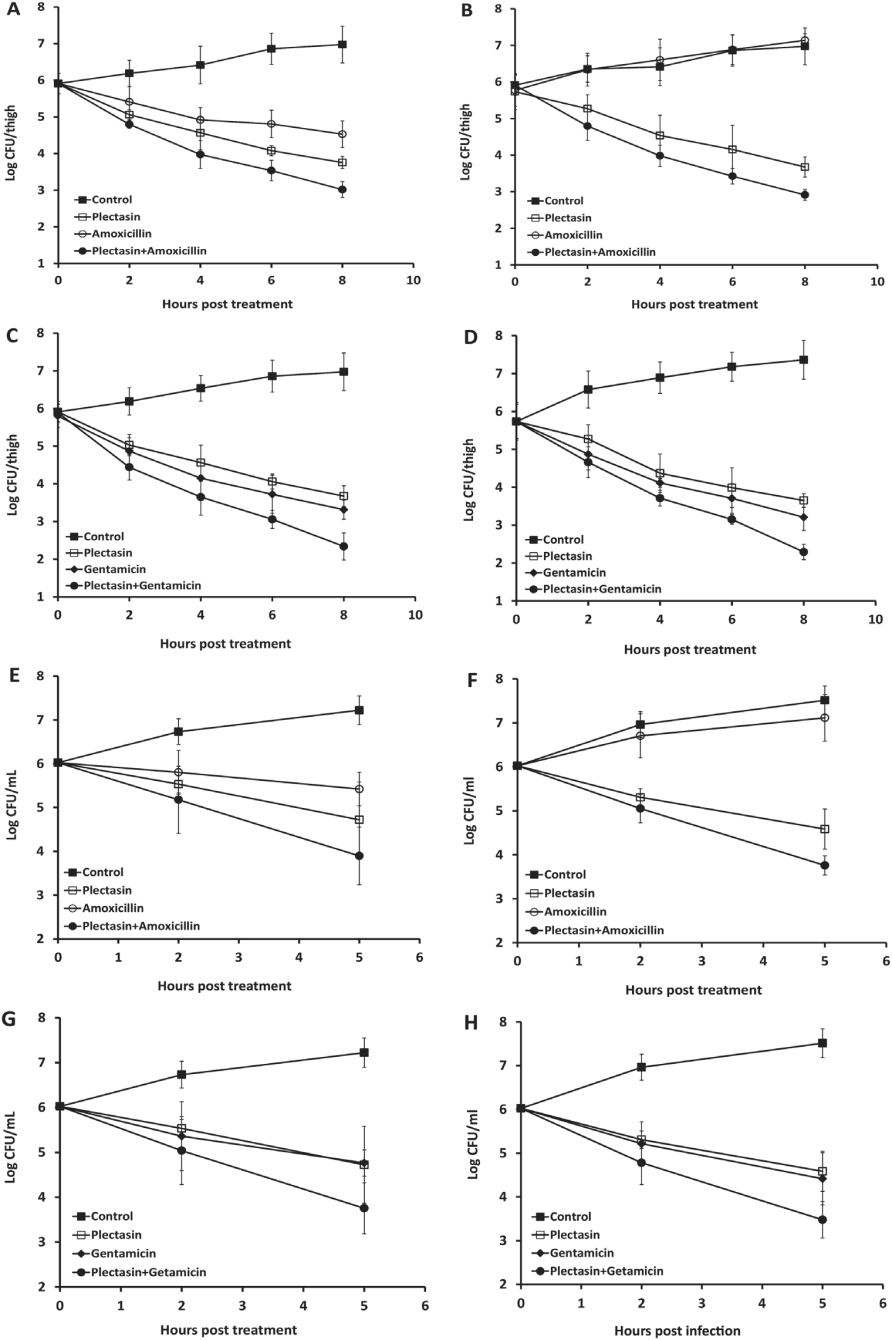
Effects of plectasin in combination with amoxicillin and gentamicin against MSSA and MRSA in a murine thigh infection model and in a mouse peritoneal infection model. In the murine thigh model, mice were infected with a strain of MSSA (t021) and a strain of MRSA (t032). Treatment was initiated 2 hours after infection with plectasin and amoxicillin against MSSA (A) and MRSA (B) and with plectasin and gentamicin against MSSA (C) and MRSA (D). Viability of the bacterial cells was determined from 6 mice for each group at 2, 4, 6 and 8 hours after treatment. In the mouse peritoneal infection model, mice were infected with the same strain of MSSA or MRSA. One hour after infection, treatment was initiated with plectasin and amoxicillin for MSSA (E) and for MRSA (F) and with plectasin and gentamicin for MSSA (G) and for MRSA (H). Bacterial counts in the peritoneal cavity were determined from 4 mice for each group at 2 and 5 hours post-treatment. The data have been repeated once.

The *in vivo* activities of plectasin combination with amoxicillin and gentamicin were studied using two murine infection models. In the well-established neutropenic mouse thigh infection model, combination of plectasin with either amoxicillin or gentamicin was tested against one MSSA and an MRSA. These strains showed FIC indices of <0.5 and significant synergistic activities in time kill curve for each of the drug combinations. Both test isolates had a plectasin MIC of 2 mg/L and gentamicin MIC of 1 mg/L; the MIC of amoxicillin for the MSSA and the MRSA was 0.5 mg/L and 512 mg/L, respectively. A range of different doses (mg/kg) of both antibiotic and plectasin was used. Treatment was performed singly or in combination after infection. The data for the combinations most effective in killing the bacteria in mouse thigh muscle or peritoneal cavity are presented. As shown in Fig. 2A, 2B, 2C and 2D, plectasin at 8 mg/kg reduced the bacterial counts in the thigh muscles and showed 2.15 log kill at 8 hour post treatment for MSSA (Fig. 2A and 2C) and 2.26 log kill for MRSA (Fig. 2B and 2D). Amoxicillin at 20 mg/kg killed 1.38 log of MSSA over an 8 hour period (Fig. 2A) and failed to control the growth of the MRSA (Fig. 2B). Plectasin in combination with amoxicillin significantly increased the kill rate against the MSSA compared to the single drug treatment (P <0.02 compared with plectasin or amoxicillin alone, n = 6) and reached 2.89 log kill at 8 hours (Fig. 2A). Similarly, combination of plectasin and amoxicillin showed significant reduction of 2.83 log of CFU counts in the mouse thigh muscles with MRSA infection compared to the single drug treatment (Fig. 2B. P <0.01 compared with plectasin or P <0.0001 with amoxicillin alone, n = 6). Gentamicin at 5 mg/kg showed bactericidal activity against both the MSSA and MRSA strains in the model (Fig. 2C and 2D) which reduced 2.79 and 2.9 log CFU counts at 8 hours, respectively. There was significant reduction of the CFU counts when gentamicin was combined with plectasin showing 3.57 and 3.82 log kill at 8 hours for both MSSA (P <0.002 compared with plectasin or P <0.01 with gentamicin alone, n = 6) and MRSA (P <0.001 compared with plectasin or P <0.02 with gentamicin alone, n = 6), respectively. Mice in both control and amoxicillin treated MRSA infection groups developed clinical signs such as leg swelling at 8 hours of post treatment. There were no clinical signs for other treatment groups with amoxicillin, gentamicin, neomycin or plectasin singly and in combination with plectasin.

In the murine peritoneal infection model, combination of plectasin with amoxicillin or gentamicin was tested against the same MSSA and MRSA strains. At 5 hours after treatment, plectasin at 8 mg/kg killed 1.3 log of the MSSA (Fig. 2E and 2G) and 1.44 log MRSA (Fig. 2F and 2H). Amoxicillin at 20 mg/kg reduced about half a log of CFU counts against the MSSA (Fig. 2E) and showed no activity against the MRSA strain (Fig. 2F). Combination of plectasin and amoxicillin exhibited 2.13 and 2.26 log kill against the MSSA (Fig. 2E) and MRSA strains (Fig. 2F), respectively. The difference between the combination and the single drug was significant for both MSSA (p<0.02 compared with plectasin or P <0.01 with amoxicillin alone, n = 4) and MRSA (P <0.02 compared with plectasin or P <0.0001 with amoxicillin alone, n = 4). As seen in Fig. 2G and 2H, gentamicin alone killed 1.26 log of the MSSA and 1.6 log of the MRSA at 5 hours. In combination with plectasin, 2.13 and 2.54 log kill was observed for both the MSSA (P <0.02 compared with plectasin or P <0.02 with gentamicin alone, n = 4) and MRSA strains (P <0.02 compared with plectasin or P <0.02 with gentamicin alone, n = 4), respectively. In both untreated control groups and the MRSA infected group treated with amoxicillin, all animals in each group developed clinical signs such as raised fur and mild immobility at 5 hours after infection. The animals in other treatment groups showed no discomfort with normal and heathy behaviors.

### Investigation of membrane potential of plectasin in combination with amoxicillin, gentamicin or neomycin

The effect of plectasin alone and in combination with amoxicillin, gentamicin or neomycin on the cytoplasmic membrane of bacterial cells was investigated. Log-phase cultures of the MSSA or MRSA strain used in the animal models were treated with the fluorescent probe DiSC3(5) which accumulates in bacterial cells and self-quenches its own fluorescence. Plectasin was added to the DiSC3(5) treated cultures at final concentrations starting from 64 mg/L to 0 mg/L. For the MSSA strain, no increase in fluorescent release was observed when the concentration of plectasin at 64 mg/L (S1A Fig.). Similarly, there was no release of fluorescence after treatment with amoxicillin, gentamicin or neomycin singly at concentrations from 64 to 2 mg/L, respectively (S1B, S1C and S1D Fig.) compared to the positive control (S1E Fig.). The cytoplasmic membrane potential was also examined when plectasin at 64 mg/L was combined with amoxicillin, gentamicin or neomycin. Combination of plectasin with amoxicillin, neomycin or gentamicin showed no impact on the fluorescence release (S1F, S1G and S1H Fig.). Similar profiles of membrane potential were observed for the MRSA strain after treatment with plectasin and the antibiotics individually or in combination (data not shown).

## Discussion

Plectasin NZ2114 is a promising synergistic agent. In our study, we found that there were significant synergistic activities when plectasin was combined with amoxicillin, penicillin, flucloxacillin, gentamicin, neomycin or amikacin *in vitro* and *in vivo*.

The panel of isolates tested represented a random collection from a range of clinical sites, the majority of which were associated with bacteraemia. Molecular analyses confirmed these isolates were genotypically diverse and included representatives of the dominant lineages of *S. aureus* recovered from humans. Plectasin NZ2114 showed significant antibacterial activity against 216 clinical isolates of both MSSA and MRSA with an MIC_50_ and MIC_90_ of 2 and 4 mg/L, respectively. On examination of the 216 clinical strains using chequerboard array analysis, synergistic activity was observed when plectasin was combined with penicillin, amoxicillin or flucloxacillin for more than 86% of both MSSA and MRSA strains. Combination of plectasin with gentamicin, neomycin and amikacin showed synergistic effects for more than 75% of each group of the strains. This positive combined effect was also confirmed using time-kill analysis which is superior to the chequerboard assay with dynamic and detailed viability measurement over time.

Bacterial resistance to antibiotics is associated with an increase in MIC to one or more antibiotics, for example, MICs of penicillin and amoxicillin for the MRSA strains tested in this study were 256 mg/L or higher. This indicates that these antibiotics are no longer effective even at the maximum tolerated dose. Here, we clearly showed that plectasin enhances the activity of penicillin, amoxicillin and flucloxacillin against both MSSA and MRSA. In particular there was a marked (2- to 512-fold) reduction in MIC for most of the MRSA strains (S2 Table). Amoxicillin at 512 mg/L was ineffective against the MRSA strain tested (Fig. 1B), however, in combination with 2 mg/L of plectasin, a rapid bactericidal effect was noted.

Aminoglycosides are potent, broad spectrum antibiotics. However, it is well known that repeated exposure of aminoglycosides, for example gentamicin, increases the risk of nephrotoxicity if used systemically or ototoxicity, particularly affecting the vestibulo-cochlear system. This limits the therapeutic usage of this class of antibiotics [26,27]. In our study, we showed that the bactericidal activities of gentamicin (one of the most commonly prescribed aminoglycosides in clinical practice) and neomycin (one of the topical antibiotics used to clear the MRSA in nose and on skin) were significantly enhanced when combined with plectasin. The concentrations of gentamicin or neomycin which produced 100% kill of log-phase MSSA or MRSA were 2 to 4 or 2 to 8 mg/L, respectively at 2 to 4 hours (data not shown). At a lower dose of 0.25 to 0.5 mg/L, gentamicin or neomycin killed the bacterial cells initially followed by a regrowth (Fig. 1). When gentamicin or neomycin at these low concentrations was combined with plectasin, 100% kill was seen at 4 hours for both MSSA and MRSA which gave the equivalent potency of the drug used singly to achieve a complete kill. Furthermore, the culture remained sterile after 48 hours, which indicated that all the bacteria were eliminated. This is significant since therapeutic effects can be achieved with lower doses of aminoglycoside, thus can dramatically reduce its side effect profile, which is particularly important where prolonged therapy is required.

The findings from our study will undoubtedly convey important positive clinical implications. Firstly, our demonstration of the efficacy of plectasin as a powerful synergistic agent strongly suggests that other similar peptides will most likely be beneficial above and beyond their direct anti-microbial properties. Secondly, synergistic combinations improve the effectiveness of antibiotics in terms of faster action and lower relapse. Thirdly, highly resistant bacteria, such as MRSA, which can be difficult to treat, may now be successfully eliminated from culture with combination therapy. Finally, combination therapy has the promising potential to reduce the therapeutic dose of antibiotics such as aminoglycosides, thus minimizing their toxic side effects.

One of the reasons for the emergence of antibiotic resistance is believed to be the poor permeability of bacterial cell envelope to antimicrobial agents [7,28,29]. Vancomycin resistance in *S. aureus* has primarily been associated with thickening of bacterial cell wall [28]. Amikacin resistance of MRSA is associated with cell wall thickening [29]. Compounds which target the cell wall or cell membrane were found to potentiate the activities of other antibiotics [14,15,17]. Such compounds also exhibited activities against stationary-phase bacteria [30–32]. Plectasin has no influence on cytoplasmic membrane potential or pore formation of the membrane [19] which was confirmed in our depolarisation study. Plectasin was found to bind to the bacterial cell wall precursor lipid II, an essential cell wall composition, to form a stoichiometric complex which resulted in the inhibition of cell wall biosynthesis [19]. This was confirmed in our study that plectasin has no bactericidal activity against stationary-phase bacteria which terminate or remodel their cell wall synthesis [33]. Further work is required to elucidate the precise mechanism underlying the activity of plectasin. Treatment with plectasin might increase the cell wall permeability against log-phase bacteria which may accelerate other antibiotics such as gentamicin and neomycin to accumulate in the cells. Gentamicin and neomycin inhibit bacterial protein synthesis and their bactericidal activities are concentration dependent [34]. Increased bactericidal activities are associated with increased bacterial intracellular concentration of the antibiotics [35,36]. The synergistic combination of plectasin and aminoglycoside antibiotics suggested that increased levels of the antibiotics inside the bacterial cells as a result of the permeabilizing effect of plectasin accelerate bacterial kill. A previous study demonstrated that vancomycin which permeabilised bacterial cell wall increased the intracellular concentration of gentamicin to 186% [37] resulting rapid death of the bacteria. It was shown that [38] streptomycin uptake was enhanced in *Enterococcus* sp. when combined with penicillin or other antibiotics which changed cell wall permeability by inhibiting cell wall synthesis. Interestingly, plectasin synergised with β-lactam antibiotics such as penicillin and amoxicillin which inhibit bacterial cell wall synthesis by predominately binding to the DD-transpeptidase and disrupt the formation of peptidoglycan cross-links [39]. The positive interaction between plectasin and β-lactam antibiotics might represent a double-hit on cell wall biosynthesis. Interestingly, in contrast to previous reports [16], in our study, there was no synergy between plectasin and vancomycin although vancomycin also binds the D-alanyl-D-alanine (D-ala-D-ala) part of the pentapeptide in Lipid II [19,40]. This difference could be due to the strains used and numbers of the strains tested. In the study of Zhang *et al* [16], only two MSSA ATCC strains were tested against the combination of plectasin with other antibiotics using the chequerboard method. In our study, more than 200 MSSA and MRSA strains, which had been isolated from hospital patients in England, were tested. In addition, we performed a detailed study by time kill curve to demonstrate the dynamic changes in reduction of CFU counts after treatment with plectasin combination. It would be prudent to conduct similar studies with strains from other geographic locations such as Asia, Australia and the United States of America to demonstrate whether the plectasin combination approach can be applied globally.

The therapeutic utility of plectasin combinations with amoxicillin and gentamicin was also examined using a mouse neutropenic thigh model and a mouse peritoneal infection model. As a potential therapeutic agent, plectasin possesses many valuable properties, it is non-toxic with a maximum tolerated dose at 125 mg/kg and has an extended stability in serum and a long half-life [16,18]. Its bactericidal activity has been reported in various animal models [20–22]. Here we demonstrate that plectasin at 8 mg/kg killed MSSA and MRSA in both mouse neutropenic thigh model and mouse peritoneal infection model. However, the combination of plectasin with amoxicillin or gentamicin improved the therapeutic activities of each single agent with significant kill of MSSA and MRSA in the mouse thigh muscles and the peritoneal cavity. Most importantly, when amoxicillin was completely ineffective against MRSA, the addition of plectasin was able to significantly reduce bacterial counts and attenuate the severity of clinical signs in the animals. Collectively, the data show the application of plectasin-antibiotic combination therapy *in vivo* offers the potential to revive the potency of conventional antibiotics, such as amoxicillin, against both MSSA and MRSA.

In conclusion, this study has shown a number of important novel mechanistic findings which we believe will be of major clinical benefit in our fight against antibiotic resistance. We have shown that plectasin, a novel agent, when used in combination with β-lactams and aminoglycosides can successfully eliminate both MSSA and MRSA *in vitro* and significantly reduced the bacterial burden *in vivo*. This combination therapy allows a lower dose of the main treatment antibiotic to be used while maintaining therapeutic potency. Most importantly, we demonstrated that rejuvenating the efficacy of existing antibiotics against resistant bacteria can potentially be applied to a range of different antibiotic classes. This early groundwork lays the foundation for further validation in clinical trials with the aim to translate combination therapy into clinical benefits for patients.

## Materials and Methods

### Bacterial strains and growth conditions

Bacterial strains used in this study included MRSA strains (115 clinical isolates from St George’s Hospital, London) and methicillin-sensitive *S. aureus* (101 clinical isolates from St George’s Hospital, London). These clinical isolates were collected from blood cultures, tissue fluid or routine screening on skin of the patients in the South West London area. Most of these strains were isolated from cases of bacteremia. Some were isolated as organ or skin colonization. The isolates were grown in nutrient broth (Oxoid) or on blood agar and trypticase soy agar (Oxoid) plates.

### *Spa* typing of MSSA and MRSA clinical isolates

*Spa* typing was performed as described previously [41]. Multi Locus Sequence Type Clonal Complex (MLST-CC) assignments were inferred based on *spa* typing data and by reference to the spa server (http://spa.ridom.de/mlst.shtml) and MLST database (http://saureus.mlst.net).

### Susceptibility tests of antibiotics against exponentially growing bacteria

The minimum inhibitory concentration (MIC) was determined in 96-well microtitre plates and *Iso-Sensitest* broth (Oxoid) in accordance with the Clinical and Laboratory Standards Institute guidelines for broth microdilution MIC [42]. Serial two-fold dilutions of antibiotics were prepared in triplicate followed by addition of a standard bacterial suspension of 1–5 × 10^5^ CFU/mL. After 24 hours at 37°C, the optical density (OD) readings were determined using an ELx800 absorbance microplate reader (BioTek). The MIC was determined as the lowest concentration of an antibiotic with similar OD reading as the control (medium only). The test antibiotics were: penicillin (powder for injection Genus Pharmaceuticals), amoxicillin (powder for injection Bowmed Limited), flucloxacillin (powder for injection Bowmed Limited) all of which were obtained from Pharmacy of St George’s hospital, London; gentamicin, neomycin and amikicin were obtained from Sigma UK; Plectacin (NZ2114) was supplied by Novozymes A/S Demark.

### Chequerboard assays to measure combination effects of drugs against log-phase bacteria

The chequerboard assay was used for the measurement of combination effects of plectasin with the antibiotics. Combinations of two drugs were prepared using 96 well plates using drug concentrations starting from two fold higher than their MIC values, then serially diluted in a twofold manner. The effects of the combinations were examined by calculating the fractional inhibitory concentration index (FICI) of each combination as follows: (MIC of drug A, tested in combination)/(MIC of drug A, tested alone) + (MIC of drug B, tested in combination)/(MIC of drug B, tested alone). The profile of the combination was defined as synergy if the FICI was ≤0.5, no interaction if the FICI was >0.5 but <4.0 and antagonism if the FICI was >4.0 [43].

### *In vitro* time kill curve tests of single drugs and combinations

Time kill curves of single drugs and drug combination were performed with a range of different concentration containing a final inoculum of 10^7^ CFU/ml of the test isolates. At 0, 2, 4, 7 and 24 hours of incubation, viability expressed as CFU/ml was determined by plating 100 μl of serial dilutions onto tryptone soya agar (Oxoid) plates or blood agar plates which were incubated at 37°C for 24 hours. The CFU was counted using aCOLyte colony counter (Synbiosis) and analyzed using the counter’s software.

### Mouse thigh infection model

Female ICR mice (five to six weeks old, body weight 24–26g) were used (Harlan UK Ltd) for the mouse thigh infection model [7]. The animal husbandry and animal care guidelines were followed according to the Animals Scientific Procedures Act, 1986 (an Act of the Parliament of the United Kingdom 1986 c. 14). The study was specifically approved by the animal ethical committee of St George’s, University of London. In order to render the mice neutropenic, two doses of cyclophosphamide (Sigma) were injected intraperitoneally 3 days (150 mg/kg) and 1 day (100 mg/kg) before the infection of the bacteria. A previous study demonstrated that this regimen of cyclophosphamide produced neutropenia in mice for 5 days [44]. Fifty microlitres of the log phase culture which contained 5 ×10^5^ bacteria was injected into right posterior thigh muscle of the mouse. After 2 hours, plectasin (8 mg/kg) and amoxicillin (20 mg/kg) or gentamicin (5 mg/kg) singly or in combination was injected intravenously into the mice. At each time point after infection and antimicrobial treatment, the thigh muscles from 6 mice for each group were aseptically removed and transferred into a 2 ml tube containing 1 ml sterile distilled water and 2 mm diameter glass beads. The thigh muscles were homogenised using a reciprocal shaker (Thermo Hybaid Ltd) for 40 seconds at 6.5 speed. The homogenates were diluted and CFU counts were performed. Untreated control mice were killed at the same time points as the treated ones.

### Mouse peritoneal infection model

Female ICR mice (five to six weeks old, body weight 24–26g) were used (Harlan UK Ltd) for the mouse peritoneal infection model [18]. The mice were infected intraperitoneally with 200 l of an overnight culture of the test isolate. After one hour, plectasin (8 mg/kg) and amoxicillin (20 mg/kg) or gentamicin (5 mg/kg) singly or in combination was injected intravenously into the mice. A group of untreated mice was included as a control. At 2 and 5 hours after treatment, 4 mice in each group were sacrificed and 1 ml sterile phosphate buffered saline (PBS) was injected intraperitoneally followed by gently massaging of the abdomen. Peritoneal fluid was sampled aseptically. The fluid was diluted and CFU counts were performed.

### Measurement of bacterial cytoplasmic membrane potential

The effect of drug treatment on bacterial cytoplasmic membrane potential was measured using a fluorescent assay with a membrane potential sensitive cyanine dye, DiSC3(5) (Dipropylthiacarbocyanine), as described previously [45]. Bacterial cells from log phase cultures were washed with PBS and resuspended with the same buffer to an optical density of 0.05 at 600 nm. The cell suspension was incubated with 0.4 μM DiSC3(5) (Sigma) until a stable (approximately 90%) reduction in fluorescence was reached as a result of DiSC3(5) uptake and quenching in the cell due to an intact membrane potential. This was followed by addition of KCl into the cell suspension at a final concentration of 100 mM to equilibrate the intracellular and external K^+^ concentrations. The treated cell suspension was placed into wells of a 96 well flat bottom fluorescence microtitre plate (Fischer Scientific UK) followed by addition of different concentrations of drugs individually or in combination in triplicate. Fluorescence was monitored using a fluorescence spectrophotometer (Glomax Multi detection system, Promega) at an excitation wavelength of 622 nm and an emission wavelength of 670 nm. The background was subtracted using a control which contained only the cells and the dye.

### Statistical analysis

The significance of differences between experimental groups was determined by Student’s t test. P values <0.05 were considered significant.

## Supporting Information

S1 Fig Determination of cytoplasmic membrane potential by plectasin, amoxicillin, gentamicin and neomycin alone or plectasin in combination with each antibiotic. Log phase MSSA culture was incubated with DiSC3(5) to a final concentration of 0.4 μM until no further quenching was detected, followed by addition of 0.1 M KCl. Plectasin, amoxicillin, gentamicin and neomycin were incubated with the cultures individually or in combination. The changes in fluorescence were monitored at various time points. A, plectasin alone. B, amoxicillin alone. C, gentamicin alone. D, neomycin alone. E, HT61 as a positive control. F, plectasin in combination with amoxicillin. G, plectasin in combination with gentamicin. H, plectasin in combination with neomycin. The data were confirmed in two independent experiments.
(EPS)

S1 Table Reduction of MICs of antibiotics in combination with plectasin against MSSA. (DOCX)

S2 Table Reduction of MICs of antibiotics in combination with plectasin against MRSA. (DOCX)
