# Supplementary material for: Combinations of β-Lactam or Aminoglycoside Antibiotics with Plectasin Are Synergistic against Methicillin-Sensitive and Methicillin-Resistant Staphylococcus aureus
Source: PLoS One. 2015 Feb 18;10(2):e0117664. doi: 10.1371/journal.pone.0117664 (PMC4333121; doi:10.1371/journal.pone.0117664)
Supplement: S1 Table — (DOCX) [file pone.0117664.s002.docx]

| \| Table S1. Reduction of MICs of antibiotics in combination with plectasin against MSSA \| \| \| \| \| --- \| --- \| --- \| --- \| \|  \| MIC (mg/L) \| \|  \| \| FIC index \| Amoxicillin \| Amoxicillin + Plectasin \| Number of strains \| \| ≤ 0.5 \| 0.25 \| 0.0625 \| 17 \| \| ≤ 0.5 \| 0.5 \| 0.0625 - 0.125 \| 20 \| \| ≤ 0.5 \| 1 \| 0.0625 - 0.25 \| 40 \| \| ≤ 0.5 \| 2 \| 0.125 - 0.5 \| 12 \| \| ≤ 0.5 \| 8 \| 2 \| 1 \| \| 0.56 -1 \| 0.0625 \| 0.0625 \| 1 \| \| 0.56 -1 \| 0.125 \| 0.0625 - 0.125 \| 2 \| \| 0.56 -1 \| 1 \| 0.5 - 1 \| 8 \| \|  \| Penicillin \| Penicillin + Plectasin \|  \| \| ≤ 0.5 \| 0.25 \| 0.031 - 0.0625 \| 16 \| \| ≤ 0.5 \| 0.5 \| 0.0625 - 0.125 \| 20 \| \| ≤ 0.5 \| 1 \| 0.0625 - 0.25 \| 36 \| \| ≤ 0.5 \| 2 \| 0.125 - 0.5 \| 17 \| \| 0.56 -1 \| 0.0625 \| 0.0625 \| 4 \| \| 0.56 -1 \| 1 \| 0.5 - 1 \| 8 \| \|  \| Flucloxacillin \| Flucloxacillin + Plectasin \|  \| \| ≤ 0.5 \| 0.25 \| 0.031 - 0.0625 \| 17 \| \| ≤ 0.5 \| 0.5 \| 0.0625 - 0.125 \| 30 \| \| ≤ 0.5 \| 1 \| 0.0625 - 0.25 \| 29 \| \| ≤ 0.5 \| 2 \| 0.125 - 0.5 \| 12 \| \| 0.56 -1 \| 0.0625 \| 0.0625 \| 5 \| \| 0.56 -1 \| 1 \| 0.5 - 1 \| 8 \| \|  \| Gentamicin \| Getamicin + Plectasin \|  \| \| ≤ 0.5 \| 0.125 \| 0.031 \| 4 \| \| ≤ 0.5 \| 0.25 \| 0.0625 \| 16 \| \| ≤ 0.5 \| 0.5 \| 0.0625 - 0.125 \| 23 \| \| ≤ 0.5 \| 1 \| 0.0625 - 0.25 \| 30 \| \| ≤ 0.5 \| 2 \| 0.0625 \| 1 \| \| ≤ 0.5 \| 8 \| 0.125 - 2 \| 2 \| \| ≤ 0.5 \| 128 \| 4 \| 1 \| \| ≤ 0.5 \| 256 \| 1 \| 2 \| \| 0.56 -1 \| 0.125 \| 0.0625 - 0.125 \| 12 \| \| 0.56 -1 \| 0.25 \| 0.125 - 0.25 \| 10 \| \|  \| Neomycin \| Neomycin + Plectasin \|  \| \| ≤ 0.5 \| 0.25 \| 0.0625 \| 12 \| \| ≤ 0.5 \| 0.5 \| 0.0625 - 0.125 \| 24 \| \| ≤ 0.5 \| 1 \| 0.0625 - 0.25 \| 29 \| \| ≤ 0.5 \| 2 \| 0.0625 - 0.5 \| 9 \| \| ≤ 0.5 \| 4 \| 0.125 - 2 \| 2 \| \| 0.56 -1 \| 0.125 \| 0.0625 - 0.125 \| 12 \| \| 0.56 -1 \| 0.25 \| 0.125 - 0. 25 \| 13 \| \|  \| Amikacin \| Amikacin + Plectasin \|  \| \| ≤ 0.5 \| 0.25 \| 0.031 - 0.0625 \| 9 \| \| ≤ 0.5 \| 0.5 \| 0.0625 - 0.125 \| 27 \| \| ≤ 0.5 \| 1 \| 0.0625 - 0.25 \| 29 \| \| ≤ 0.5 \| 2 \| 0.0625 - 0.5 \| 5 \| \| ≤ 0.5 \| 4 \| 0.125 - 2 \| 7 \| \| 0.56 -1 \| 0.125 \| 0.0625 - 0.125 \| 12 \| \| 0.56 -1 \| 0.25 \| 0.125 - 0. 25 \| 12 \| \|  \| Vancomycin \| Vancomycin + Plectasin \|  \| \| 0.56 -1 \| 0.5 \| 0.5 \| 2 \| \| 0.56 -1 \| 1 \| 0.5 - 1 \| 43 \| \| 0.56 -1 \| 2 \| 1 - 2 \| 56 \| |
| --- | --- | --- | --- | --- | --- | --- | --- | --- | --- | --- | --- | --- | --- | --- | --- | --- | --- | --- | --- | --- | --- | --- | --- | --- | --- | --- | --- | --- | --- | --- | --- | --- | --- | --- | --- | --- | --- | --- | --- | --- | --- | --- | --- | --- | --- | --- | --- | --- | --- | --- | --- | --- | --- | --- | --- | --- | --- | --- | --- | --- | --- | --- | --- | --- | --- | --- | --- | --- | --- | --- | --- | --- | --- | --- | --- | --- | --- | --- | --- | --- | --- | --- | --- | --- | --- | --- | --- | --- | --- | --- | --- | --- | --- | --- | --- | --- | --- | --- | --- | --- | --- | --- | --- | --- | --- | --- | --- | --- | --- | --- | --- | --- | --- | --- | --- | --- | --- | --- | --- | --- | --- | --- | --- | --- | --- | --- | --- | --- | --- | --- | --- | --- | --- | --- | --- | --- | --- | --- | --- | --- | --- | --- | --- | --- | --- | --- | --- | --- | --- | --- | --- | --- | --- | --- | --- | --- | --- | --- | --- | --- | --- | --- | --- | --- | --- | --- | --- | --- | --- | --- | --- | --- | --- | --- | --- | --- | --- | --- | --- | --- | --- | --- | --- | --- | --- | --- | --- | --- | --- | --- | --- | --- | --- | --- | --- | --- | --- | --- | --- | --- | --- | --- | --- | --- | --- | --- | --- | --- | --- | --- | --- | --- | --- | --- | --- | --- | --- | --- | --- | --- | --- | --- | --- | --- |
