# Supplementary material for: Combinations of β-Lactam or Aminoglycoside Antibiotics with Plectasin Are Synergistic against Methicillin-Sensitive and Methicillin-Resistant Staphylococcus aureus
Source: PLoS One. 2015 Feb 18;10(2):e0117664. doi: 10.1371/journal.pone.0117664 (PMC4333121; doi:10.1371/journal.pone.0117664)
Supplement: S2 Table — (DOCX) [file pone.0117664.s003.docx]

| Table S2. Reduction of MICs of antibiotics in combination with plectasin against MRSA   \|  \| MIC (mg/L) \| \|  \| \| --- \| --- \| --- \| --- \| \| FIC index \| Amoxicillin \| Amoxicillin + Plectasin \| Number of strains \| \| ≤ 0.5 \| 256 \| 0.5 \| 4 \| \|  \|  \| 1 \| 16 \| \|  \|  \| 4 \| 12 \| \|  \|  \| 8 \| 2 \| \|  \|  \| 64 \| 2 \| \| ≤ 0.5 \| 512 \| 0.15 \| 3 \| \|  \|  \| 0.5 \| 5 \| \|  \|  \| 1 \| 4 \| \|  \|  \| 2 \| 10 \| \|  \|  \| 8 \| 4 \| \|  \|  \| 16 \| 3 \| \|  \|  \| 128 \| 2 \| \| ≤ 0.5 \| >512 \| 0.5 \| 9 \| \|  \|  \| 2 \| 7 \| \|  \|  \| 4 \| 8 \| \|  \|  \| 8 \| 9 \| \|  \|  \| 64 \| 1 \| \| 0.56 -1 \| 256 \| 128 - 256 \| 6 \| \| 0.56 -1 \| 512 \| 256 - 512 \| 8 \| \|  \| Penicillin \| Penicillin + Plectasin \|  \| \| ≤ 0.5 \| 128 \| 0.5 \| 6 \| \|  \|  \| 2 \| 12 \| \|  \|  \| 4 \| 4 \| \|  \|  \| 8 \| 7 \| \|  \|  \| 16 \| 4 \| \|  \|  \| 32 \| 2 \| \| ≤ 0.5 \| 256 \| 0.5 \| 4 \| \|  \|  \| 1 \| 6 \| \|  \|  \| 2 \| 17 \| \|  \|  \| 4 \| 2 \| \|  \|  \| 64 \| 2 \| \| ≤ 0.5 \| 512 \| 1 \| 12 \| \|  \|  \| 2 \| 3 \| \|  \|  \| 4 \| 8 \| \|  \|  \| 8 \| 5 \| \|  \|  \| 32 \| 1 \| \|  \|  \| 64 \| 6 \| \|  \|  \| 128 \| 1 \| \| 0.56 -1 \| 128 \| 64 -128 \| 7 \| \| 0.56 -1 \| 256 \| 128 -256 \| 6 \| \|  \| Flucloxacillin \| Flucloxacillin + Plectasin \|  \| \| ≤ 0.5 \| 128 \| 0.5 \| 6 \| \|  \|  \| 1 \| 12 \| \|  \|  \| 2 \| 2 \| \|  \|  \| 4 \| 4 \| \|  \|  \| 8 \| 3 \| \|  \|  \| 32 \| 3 \| \| ≤ 0.5 \| 256 \| 0.5 \| 6 \| \|  \|  \| 1 \| 15 \| \|  \|  \| 2 \| 8 \| \|  \|  \| 4 \| 2 \| \|  \|  \| 64 \| 4 \| \| ≤ 0.5 \| 512 \| 1 \| 9 \| \|  \|  \| 2 \| 10 \| \|  \|  \| 4 \| 4 \| \|  \|  \| 8 \| 4 \| \|  \|  \| 16 \| 3 \| \|  \|  \| 64 \| 4 \| \|  \|  \| 128 \| 3 \| \| 0.56 -1 \| 128 \| 64 -128 \| 7 \| \| 0.56 -1 \| 256 \| 128 -256 \| 6 \| \|  \| Gentamicin \| Getamicin + Plectasin \|  \| \| ≤ 0.5 \| 1 \| 0.0625 - 0.25 \| 50 \| \| ≤ 0.5 \| 2 \| 0.0625 - 0.5 \| 39 \| \| 0.56 -1 \| 1 \| 0.5 - 1 \| 12 \| \| 0.56 -1 \| 2 \| 1 - 2 \| 14 \| \|  \| Neomycin \| Neomycin + Plectasin \|  \| \| ≤ 0.5 \| 1 \| 0.0625 - 0.25 \| 22 \| \| ≤ 0.5 \| 2 \| 0.0625 - 0.5 \| 23 \| \| ≤ 0.5 \| 4 \| 0.25 - 1 \| 20 \| \| ≤ 0.5 \| 8 \| 0.25 - 2 \| 23 \| \| 0.56 -1 \| 1 \| 1 to 2 \| 12 \| \| 0.56 -1 \| 4 \| 2 to 4 \| 15 \| \|  \| Amikacin \| Amikacin + Plectasin \|  \| \| ≤ 0.5 \| 1 \| 0.0625 - 0.25 \| 27 \| \| ≤ 0.5 \| 2 \| 0.0625 - 0.5 \| 19 \| \| ≤ 0.5 \| 4 \| 0.25 - 1 \| 22 \| \| ≤ 0.5 \| 8 \| 0.25 - 2 \| 20 \| \| 0.56 -1 \| 1 \| 1 - 2 \| 15 \| \| 0.56 -1 \| 4 \| 2 - 4 \| 12 \| \|  \| Vancomycin \| Vancomycin + Plectasin \|  \| \| 0.56 -1 \| 1 \| 0.5 - 1 \| 55 \| \| 0.56 -1 \| 2 \| 1 - 2 \| 60 \| |
| --- | --- | --- | --- | --- | --- | --- | --- | --- | --- | --- | --- | --- | --- | --- | --- | --- | --- | --- | --- | --- | --- | --- | --- | --- | --- | --- | --- | --- | --- | --- | --- | --- | --- | --- | --- | --- | --- | --- | --- | --- | --- | --- | --- | --- | --- | --- | --- | --- | --- | --- | --- | --- | --- | --- | --- | --- | --- | --- | --- | --- | --- | --- | --- | --- | --- | --- | --- | --- | --- | --- | --- | --- | --- | --- | --- | --- | --- | --- | --- | --- | --- | --- | --- | --- | --- | --- | --- | --- | --- | --- | --- | --- | --- | --- | --- | --- | --- | --- | --- | --- | --- | --- | --- | --- | --- | --- | --- | --- | --- | --- | --- | --- | --- | --- | --- | --- | --- | --- | --- | --- | --- | --- | --- | --- | --- | --- | --- | --- | --- | --- | --- | --- | --- | --- | --- | --- | --- | --- | --- | --- | --- | --- | --- | --- | --- | --- | --- | --- | --- | --- | --- | --- | --- | --- | --- | --- | --- | --- | --- | --- | --- | --- | --- | --- | --- | --- | --- | --- | --- | --- | --- | --- | --- | --- | --- | --- | --- | --- | --- | --- | --- | --- | --- | --- | --- | --- | --- | --- | --- | --- | --- | --- | --- | --- | --- | --- | --- | --- | --- | --- | --- | --- | --- | --- | --- | --- | --- | --- | --- | --- | --- | --- | --- | --- | --- | --- | --- | --- | --- | --- | --- | --- | --- | --- | --- | --- | --- | --- | --- | --- | --- | --- | --- | --- | --- | --- | --- | --- | --- | --- | --- | --- | --- | --- | --- | --- | --- | --- | --- | --- | --- | --- | --- | --- | --- | --- | --- | --- | --- | --- | --- | --- | --- | --- | --- | --- | --- | --- | --- | --- | --- | --- | --- | --- | --- | --- | --- | --- | --- | --- | --- | --- | --- | --- | --- | --- | --- | --- | --- | --- | --- | --- | --- | --- | --- | --- | --- | --- | --- | --- | --- | --- | --- | --- | --- | --- | --- | --- | --- | --- | --- | --- | --- | --- | --- | --- | --- | --- | --- | --- | --- | --- | --- | --- | --- | --- | --- | --- | --- | --- | --- | --- | --- | --- | --- | --- | --- | --- | --- | --- |
